# Editorial

**Published:** 1875-01

**Authors:** 


### Editorial.

The Concours for the position of Lecturer on Obstetrics in the Spring Faculty of Rush Medical College which was opened at the College on the evening of Tuesday, Dec. 8th, and continued on the 9th and 10th, was finally concluded upon the evening of Friday, Dec. 11th, in a manner as unexpected as gratifying to all concerned.

The announcement of the Concours had excited more general attention and interest than those which had preceded it, as it was recognized as an expression of the determination of the Faculty to fill up vacancies in their ranks by the test of merit alone.

Fourteen applications were made for admission to the Concours ; four of these, however, were subsequently withdrawn, leaving but ten who actually competed for the position. To these, subjects were assigned, and one week granted for preparation of the lectures, which were delivered in the following order:

TUESDAY.

DR. C. W. EARLE — Phlegmasia Dolens.
" C. T. FENN — Obstetric Forceps.
" J. S. KNOX — Histological Anatomy and Functions of Placenta.

WEDNESDAY.

DR. W. C. LYMAN — Abortion.
" A. VON MANSFELDE — Retained Placenta.
" W. P. PEIRCE — Conception and Early Development of the Ovum.

THURSDAY.

DR. G. F. ROBERTS — Unavoidable Hæmorrhage.
" A. K. STEELE — Eclampsia Parturientium.
" E. W. SAWYER — Post Partum Hæmorrhage.
" G. D. WILBUR — Puerperal Mania.

Thus far the Concours had proved a most agreeable surprise to the entire audience, composed of the professional men of the city and students, and it is extremely doubtful if as great a number of lectures of so high a degree of excellence, evincing so much intellectual power and professional erudition, have ever been delivered upon a similar occasion in this country.

In Dr. Earle we recognized the dignity and power of the experienced teacher, who, while already holding a full professorship in another college, still considered a lectureship in Rush Medical College a prize worth striving for.

Dr. Fenn, also a lecturer of some years' standing, exhibited in his lecture upon the Obstetric Forceps, the evidences of patient and laborious study, and the pluck which never recognizes defeat.

The lecture of Dr. Knox was a masterpiece of scientific accuracy, couched in "English pure and undefiled," delivered with elegance of style and ease of manner, which made listening a pleasure.

Dr. Lyman presented the subject of abortion to his auditors in a most earnest and impressive manner, characterized by the evidences of profound thought and careful study.

Dr. Von Mansfelde's illustrations of the modes of "placental retention" were graphic and instructive, and were closed with a graceful compliment to his Alma Mater.

Dr. W. P. Peirce, in his demonstration of "Conception and the Early Development of the Ovum," betrayed in the manner the experienced teacher, and in the matter the skilled practitioner who had "thought out for himself" many of the knotty problems in physiology as well as practical medicine. His lecture was well illustrated by some excellent original diagrams, and illuminated with flashes of humor which won for him many votes among the benches.

Dr. G. F. Roberts, one of Rush's youngest alumni, gratified his old teachers by demonstrating practically, the excellence of their instructions, in the admirable manner in which he discussed the subject of "Unavoidable Hæmorrhage," and satisfied the Faculty that there is among the old pupils a powerful reserve force to sustain the reputation of the old school unimpaired.

Dr. A. K. Steele's thorough practical familiarity with his subject, "Eclampsia Parturientium" and especially with its symptomatology and therapeusis, soon proved to all that he was to be one of the winners, and its continuation and close left that impression strengthened in the minds of many of his auditors.

Dr. E. W. Sawyer elucidated the subject of "Post Partum Hæmorrhage" in a manner which surprised and delighted his hearers. The comprehension and accuracy of his knowledge, the clearness of his style, and the precision of his system, displayed the accomplished scholar and the trained thinker.

Dr. G. D. Wilbur had the distinction of closing this stage of the Concours by an admirable lecture on "Puerperal Mania," in which, although last, he proved himself by no means least.

But the Concours was not yet over, the struggle not yet terminated. Out of the ten competitors four were selected "pares inter primos," and between these the Faculty found it impossible to decide to whom the prize should be awarded, and the application of still another and a more severe test became necessary to determine the victory.

Titles of subjects were written upon separate slips of paper, placed in a hat, and from this hat the four selected at the previous trial were required to draw each in his turn a slip, and announcing the inscribed title, to proceed at once to lecture upon the subject thus assigned him by dame Fortune.

The subjects thus assigned were—

To Dr. J. S. Knox — Rupture of the Uterus.
To Dr. E. W. Sawyer — Anatomy of the Female Pelvis and its Deformities.
To Dr. G. F. Roberts — Inversion of the Uterus.
To Dr. A. K. Steele — Labor in Face Presentations.

There are few physicians, however great their skill, or however extensive or prolonged their experience, who would not shrink from an ordeal so severe, and still fewer who, having made the essay, would not utterly fail. It was truly an "experimentum crucis," and no small meed of praise should be theirs who, having been found worthy, even dared the trial.

That three should fail, no, not fail, for such men never fail—should not succeed, was inevitable, as there was but one prize; had there been four, the success of each and every one had been secure.

Out of this trial two came unscathed, and the only regret felt and expressed by the judges in awarding the lectureship to Dr. Sawyer, was the necessity of withholding it from Dr. Knox.

On several occasions this Journal has taken the liberty to animadvert upon the low estimate at which the medical profession is held by the civil—we should rather say the political—authorities, and also to some of the causes which have induced this result. One of these was indicated in our pages in the early portion of the past year, to be the unseemly haste manifested by certain physicians to rush into the arena of politics and to struggle for the prizes of petty offices which, in the majority of instances, are only the first steps in the direction of professional demoralization.

There are certain offices in the gift of the county and municipal authorities which can only with propriety be filled by medical men. We refer to those of City and County Physicians, Sanitary Superintendent, and Coroner. The first three of these are so filled, and we believe well filled, fortunately for the public, although by no means fortunately for the incumbents, who find themselves in positions involving grave professional and civic responsibilities and very arduous labor, with authority by no means adequate, and compensation totally inadequate thereto. The duties of the office of Coroner can be satisfactorily filled by a thoroughly educated physician only, and by no one else.

No degree of intelligence or general culture, no amount of energy or fidelity, will compensate for the technical education acquired by physicians only—an education enabling them to recognize and appreciate a class of facts unappreciable otherwise.

The office of Coroner, rightly administered, might and should constitute one of the corner stones of the whole system of criminal jurisprudence, instead of, as hitherto, a butt for the shafts of well merited ridicule, till "crowner's-quest law" has come to be a synonym of burlesque from the days of Shakespeare until now.

The verdict of a Coroner's jury as at present constituted, consists, not of the logical conclusion of twelve intelligent men, based upon the *facts* submitted to their consideration, in the evidence set before them, but the *opinion* of the county physician or of some other medical gentleman who may have some knowledge of the case. Nine times out of ten, the "intelligent jury" gathered from the neighboring bar-rooms and express wagons, render as their verdict these *opinions* verbatim—from utter inability, through ignorance, to comprehend the facts, and to come to any independent conclusion.

Just so long as the authorities offer and pay for medical services compensation about equivalent to what is paid for the simplest forms of unskilled labor, and make the office of Coroner the reward of political services, just so long will the operations of public justice be completely obstructed in this direction, and many a crime remain concealed, which under a more enlightened and liberal system of medico-legal investigation would be brought to light. H.

***Book-keeping.***

Those of our readers who have done us the kindness to borrow from our exchange files the following Journals, will add to the obligation by returning the same at their earliest convenience:

Boston Medical and Surgical Journal, Sept., '73, July, Aug., Sept., Oct., '74.

Philadelphia Medical and Surgical Reporter, vol. xxx, Nos. 22, 23, 24; vol. xxxi, Nos. 12, 14, 21, 22.

Atlanta Medical and Surgical Journal, May and September, '74.

American Practitioner, Feb., May, Aug., '74.

New York Medical Journal, May, July, Aug., '74.

Philadelphia Medical Times, Nos. 105, 131, 141, 148, 152, 155, 160, 161.

---

***The Illinois Charitable Eye and Ear Infirmary.***

This institution now occupies its new building, corner of Adams and Peoria Streets, Chicago. The edifice, 105 feet long by 47 feet wide, consisting of a basement and four stories with a mansard roof, presents a most attractive appearance. It is an ornament to the portion of the city in which it is situated.

The accommodations for the treatment and comfort of patients are equaled by those of no similar institution in the country.

From the Seventeenth Annual Report we learn that during the past year 1,012 patients were treated gratuitously, making an aggregate of 10,620 patients that have received the benefits of the Infirmary since 1858, the year of its foundation.

The following is a form of certificate which will admit any poor patient residing in Illinois to the Infirmary, or gratuitous treatment and board:

This is to Certify, that ———, of the Town of ———, County of ———, State of Illinois, is absolutely without means to pay for his (*or* her) board or treatment at the Illinois Charitable Eye and Ear Infirmary.

Form of certificate which will admit any poor patient to the Infirmary for gratuitous treatment:

This is to Certify, that ———, of the Town of ———, County of ———, State of———, is in indigent circumstances, and can pay his (*or* her) board alone, but not for treatment, at the Illinois Charitable Eye and Ear Infirmary.

Either form of certificate must be signed by some respectable physician, or Supervisor, or Clerk of the County Court, where the patient resides.

Neither of the above certificates should in any case be given to a patient unless he be destitute of means.

The Dispensary of the Infirmary is open daily from 2 to 2½ P. M., for such poor patients, not boarding at the Infirmary, as may require treatment.

